# Neuroprotective Mechanisms of Paeoniflorin in Parkinson’s Models: Involvement of BDNF-Dependent PI3K/Akt and ERK/CREB Pathways

**DOI:** 10.3390/cimb48090944

**Published:** 2026-09-16

**Authors:** Chang Jin, Yue Zhang, Bing Li, Zhifeng Cheng, Meizhu Zheng, Kai Song, Yongxing Ai

**Affiliations:** 1 College of Life Science, Changchun Normal University, Changchun 130032, China; j1504408478@163.com (C.J.); 15942907284@163.com (Y.Z.); 18544359016@163.com (B.L.); czf20170726@163.com (Z.C.); 2 The Central Laboratory, Changchun Normal University, Changchun 130032, China; 3 College of Animal Science, Jilin University, Changchun 130062, China; aiyx@jlu.edu.cn

**Keywords:** Parkinson’s disease, paeoniflorin, neuroprotection, PI3K/Akt, ERK/CREB

## Abstract

Paeoniflorin, a bioactive monoterpene glycoside derived from *Paeonia lactiflora*, has shown neuroprotective effects primarily associated with the promotion of brain-derived neurotrophic factor secretion. This study aims to elucidate the therapeutic potential of paeoniflorin in models of Parkinson’s disease and to investigate the role of the BDNF-mediated PI3K/Akt and ERK1/2/p90RSK/CREB signaling pathways. Notably, paeoniflorin improved motor function, reduced dopamine catabolism, and attenuated dopaminergic neuronal loss in PD mice by regulating dopamine catabolism and inhibiting turnover, indicating dopaminergic protection. In vitro, paeoniflorin pretreatment enhanced cell viability, reduced LDH release, suppressed ROS and intracellular Ca^2+^ overload, stabilized mitochondrial membrane potential, and attenuated MPP^+^-induced apoptosis. A key innovation is the dual activation and coordinated activation of the two BDNF-dependent pathways: LY294002 abolished paeoniflorin-induced BDNF/PI3K/Akt activation, while PD98059 and ERK1/2 siRNA specifically suppressed paeoniflorin-triggered phosphorylation of ERK1/2, p90RSK, and CREB. Ultimately, paeoniflorin exerts robust neuroprotection against MPTP/MPP^+^ neurotoxicity by synergistically activating BDNF-downstream PI3K/Akt and ERK/CREB axes. This delineation of a dual-pathway mechanism provides novel insight into the pharmacological action of paeoniflorin and supports its potential as a multifaceted therapeutic agent for Parkinson’s disease.

## 1. Introduction

Parkinson’s disease (PD) is a common neurodegenerative disorder in the middle-aged and elderly [1,2,3]. Although clinical drugs including levodopa and dopamine agonists relieve motor symptoms of PD, they cannot block pathological progression [4,5]. Current treatments also cause obvious adverse reactions, such as mental disorders and gastrointestinal injury [6,7]. Therefore, it is necessary to develop effective and safe neuroprotective agents to delay PD progression. Accumulating studies have confirmed the neuroprotective effects of traditional Chinese medicines in neurodegenerative diseases [8,9,10], indicating that TCM is a reliable resource for anti-PD research.

Paeoniflorin (PF) is the main active component of *Paeonia lactiflora* and exerts multiple biological functions [11]. It regulates the nervous and immune systems and affects tumor cell activities [11]. Previous studies have demonstrated that PF improves behavioral deficits and protects dopaminergic neurons in MPTP-induced PD models, supporting its potential neuroprotective effects against PD-related neurodegeneration [12]. However, these studies primarily focused on the pharmacological efficacy of PF in animal models, while the molecular mechanisms underlying its neuroprotective actions remain to be fully elucidated. In particular, whether PF mediates neuroprotection through specific intracellular signaling networks associated with neuronal survival and synaptic plasticity remains unclear. Moreover, PF is known to exhibit poor blood–brain barrier (BBB) permeability, raising important questions regarding whether its neuroprotective actions are mediated via direct central nervous system (CNS) targeting or through indirect mechanisms such as gut microbiota modulation, bioactive metabolite conversion, or peripheral anti-inflammatory effects [13]. Despite these pharmacokinetic considerations, PF retains significant value as a tool compound for mechanistic investigation; its established safety profile, structural simplicity, and documented bioactivity make it an ideal candidate for elucidating the intrinsic cellular signaling pathways that mediate neuroprotection. Such mechanistic insights are essential not only for understanding the therapeutic potential of PF itself but also for guiding the development of optimized derivatives with improved pharmacokinetic properties. Nevertheless, the pharmacological effects of PF on PD and its underlying mechanisms remain inadequately understood.

Growing evidence has confirmed that PI3K/Akt pathway dysfunction is closely correlated with PD pathogenesis [14]. BDNF binds to TrkB to activate the downstream PI3K/Akt pathway and protect neurons. In addition, BDNF-mediated signaling promotes ERK/p90RSK phosphorylation and CREB activation in neurons [15,16,17,18,19]. Phosphorylated CREB binds to the BDNF promoter and regulates its transcription and expression [19], thus mediating neuroplasticity and neuroprotection. The BDNF/ERK/p90RSK and PI3K/Akt/CREB signaling pathways are, therefore, critical targets for the prevention and treatment of PD.

Madopar, a combined preparation of levodopa and benserazide hydrochloride, is a classic dopamine replacement drug for PD, which was used as the positive control in this study [20,21].

**Figure 1:**
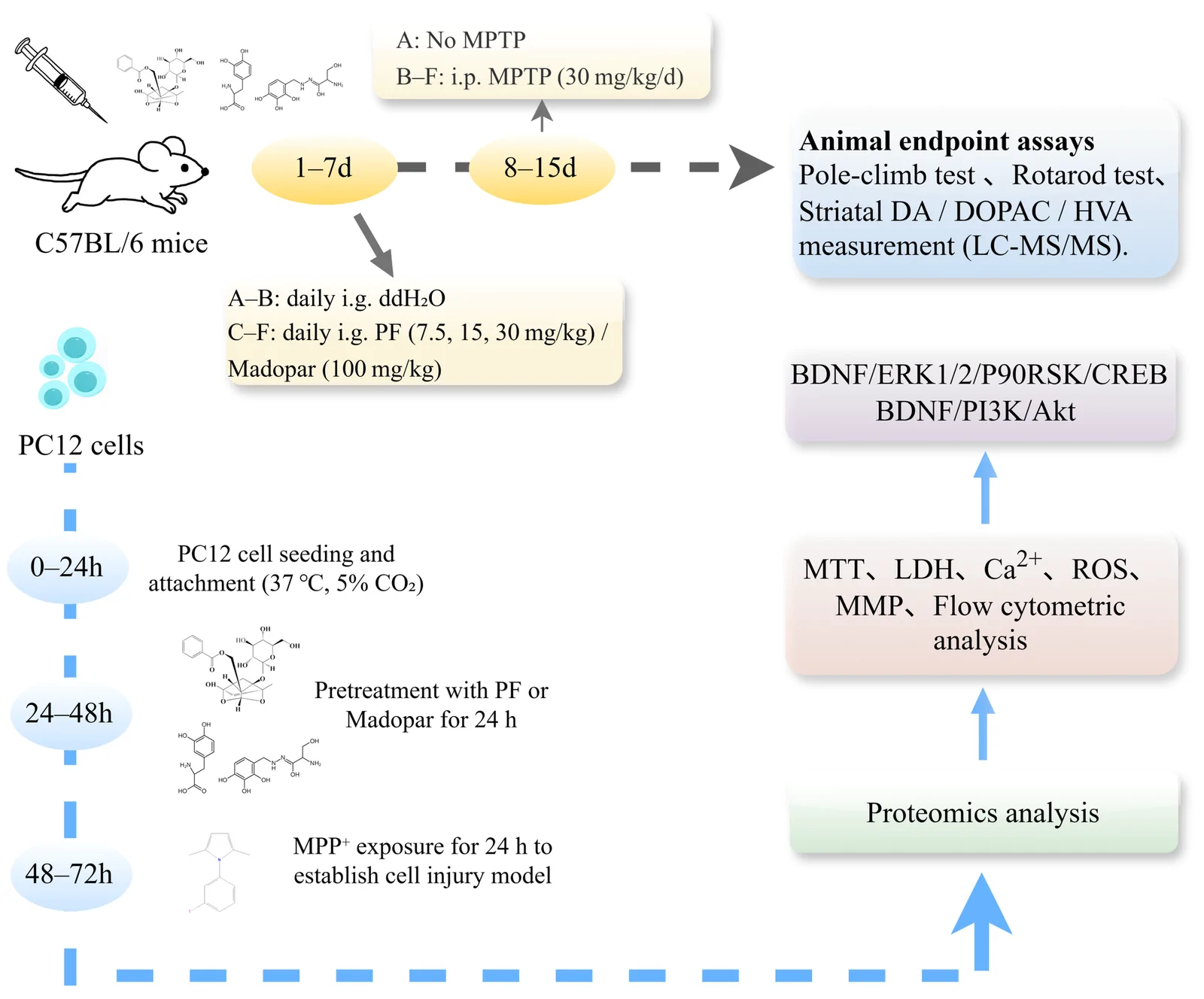
The experimental flow chart of the protective effect exerted by PF in PD.

Our previous study provided in vivo evidence that paeoniflorin (PF) protects nigral dopaminergic neurons against MPTP-induced neurodegeneration, as evidenced by preservation of TH-positive neurons in the substantia nigra [12,20]. These findings established the anti-Parkinsonian activity of PF at the phenotypic level. However, the cellular and molecular mechanisms underlying this protective effect remain insufficiently understood. Therefore, the present study was designed to extend these previous phenotypic observations by systematically investigating the molecular basis of PF-mediated neuroprotection. A limited in vivo validation experiment was included to support the previously reported protective phenotype, while the major focus was placed on integrative proteomic analysis and subsequent cellular and molecular validation. Through this approach, we sought to identify key signaling cascades that may mediate the neuroprotective actions of PF at the molecular level. The complete research process is illustrated in Figure 1.

## 2. Materials and Methods

### 2.1. Reagents

Paeoniflorin (PF, CAS: 23180-57-6, purity ≥ 98%) was purchased from Shanghai Yuanye Bio-Technology (Shanghai, China). MPTP, MPP^+^, SDS, DTT, Edaravone and Lipofectamine 2000 were obtained from Sigma-Aldrich (St. Louis, MO, USA). Madopar was supplied by Shanghai Roche Led. All primary and secondary antibodies were purchased from Abcam (Cambridge, UK), including anti-β-Actin (ab8227), anti-PI3K p85α (ab191606), anti-phospho-PI3K p85α (Y467)/p55 (Y199) (ab278545), anti-pan-AKT (ab32505), anti-phospho-AKT (S473) (ab81283), anti-Bcl-2 (ab32124), anti-Bax (ab32503), anti-cleaved-Caspase-3 (ab2302), anti-ERK1/2 (ab184699), anti-phospho-ERK1/2 (T202/Y204) (ab214362), anti-p90RSK (ab32114), anti-phospho-p90RSK (S380) (ab32203), anti-CREB1 (ab220797), anti-phospho-CREB (S133) (ab220798), and anti-BDNF (ab108319). HRP-conjugated secondary antibodies included goat anti-rabbit IgG (ab60295G-HRP) and goat anti-rat IgG (ab60120G-HRP). ERK1/2 siRNA was obtained from Genepharma (Shanghai, China). The ERK1/2 inhibitor PD98059 (Calbiochem, San Diego, CA, USA) and PI3K inhibitor LY294002 (Cell Signaling Technology, Danvers, MA, USA) were used in this study.

### 2.2. Statement of Ethics

All experimental procedures were approved by the Institutional Animal Care and Use Committee of Changchun Normal University (IACUC Approval No. 2024003) and were performed following national guidelines for animal care. Additionally, every procedure was carried out in compliance with all applicable rules and laws.

### 2.3. Animal Behavioral Assessment

For the purpose of this investigation, 8-week-old adult male C57BL/6 mice (body weight: 22–26 g) were utilized, operating under license SCXK (JING)-2005-0013. Mice were housed under standard laboratory housing conditions: 12 h light–dark cycle, constant temperature (22 ± 2 °C) and humidity (50 ± 5%), with free access to food and water. Each cage containing 4–5 mice was defined as one experimental unit. A total of 180 mice were enrolled in this study. Sample size was determined according to our approved animal-ethics protocol, preliminary experiments and published in vivo PD-related studies to achieve adequate statistical power. Animals were randomly allocated to five distinct groups by random-number table. Three independent experimental repeats were performed. Behavioral tests were performed by investigators blinded to group assignment. No pre-defined exclusion criteria were applied; no animals died or were excluded prior to endpoint analysis. Mice were intragastrically administered with paeoniflorin at doses of 7.5, 15 and 30 mg/kg once daily for 14 consecutive days. These dosage regimens were selected with reference to previous pharmacological and pharmacokinetic studies of paeoniflorin [22]. After behavioral evaluation, mice were anesthetized with pentobarbital sodium before sample collection. Urine samples were collected, and brain tissues and serum were harvested. Mice were sacrificed by cervical dislocation. Animal carcasses were subjected to centralized harmless treatment after experiments. For each group, 12 mice were included per biological replicate. All in vitro assays were performed with at least three independent biological replicates and corresponding technical replicates.

#### 2.3.1. Pole-Climb Test

The pole-climb test was applied to evaluate bradykinesia and motor coordination of mice. A vertical wooden pole with rough surface (diameter 1 cm, height 50 cm) was fixed upright. Each mouse was placed head-down at the top of the pole, and the total time taken for the mouse to climb down to the bottom ground was recorded as pole-climb latency. The maximum observation time was limited to 180 s. Every mouse received three independent tests each day, and the average value of the three trials was calculated for statistical analysis [23].

#### 2.3.2. Rotarod Test

Examination involved positioning the mice atop a revolving rod, subsequently recording the duration from the rod’s initial rotation until the point when the mice disembarked. Each testing interval was firmly set at 180 s, and the daily average was computed from three separate trials [24].

### 2.4. Measurement for the Striatum-Based DA and Its Metabolite Levels by LC-MS-MS

Following the final behavioral assessment, six mice per group were decapitated at a 72 h interval. The striatum was dissected, hemispherically divided, and flash-frozen. Each tissue sample was transferred to a pre-chilled tube, subjected to gravimetric measurement, and homogenized in ice-cold methanol at a 1:4 (*w*/*v*) ratio. The homogenate was then centrifuged (14,000× *g*, 20 min, 4 °C), and the supernatant was collected for subsequent dopamine quantification using LC-MS/MS, as previously described [25].

LC-MS/MS analysis was conducted using a Waters Acquity UPLC system (Milford, MA, USA) interfaced with a Micromass Quattro Premier XE tandem quadrupole mass spectrometer (Manchester, UK). Separation was achieved on a Thermo C18 column (4.6 mm × 250 mm, 5 μm, SN: USCL0208940, Thermo electron corporation, MA, USA) at ambient temperature. The mobile phase, comprising 0.1% formic acid and acetonitrile (92:8, *v*/*v* for DA; 82:18, *v*/*v* for metabolites), was filtered (0.2 μm) and not recirculated. The flow rate was set to 0.3 mL/min with a post-column split ratio of 4:1. Electrospray ionization was employed, with DA being monitored in positive ion mode and DOPAC/HVA in negative ion mode.

### 2.5. Cell Culture and Treatment

PC12 cells, procured from the Chinese Academy of Sciences situated in Shanghai, underwent cultivation in DMEM, supplemented with 10% fetal bovine serum, as well as 100 U/mL each of penicillin and streptomycin, and were maintained in a stable environment of 37 °C, involving 5% CO_2_ and 95% ambient air. For experimental purposes, cells experiencing logarithmic growth phase were selected. Exposure of PC12 cells to PF was systematically carried out at various concentrations ranging from 0 to 300 μM over a period of 24 h. Subsequent operations included executing the MTT test to detect any potential cytotoxicity of PF. The in vitro PD model was formulated through treating the PC12 cells with 500 μM MPP^+^ for an approximate duration of 24 h. Prior to the treatment with MPP^+^, variable concentrations of PF (25, 50, 100 μM), or alternatively Madopar (50 μg/mL), were administered to the cells 48 h in advance, following which the PC12 cells were utilized for successive experiments.

### 2.6. Cell Viability

Evaluation of cell viability was conducted utilizing the MTT assay. Following a 24 h period of drug exposure, MTT solution (10 μL, 0.5 mg/mL) was introduced to every individual well, followed by an incubation period of 4 h at a stable temperature of 37 °C. Thereafter, the culture medium was conscientiously removed, and DMSO (100 μL) was added to facilitate the dissolution of formazan. In the concluding step, an enzyme-labeling instrument, specifically the Flex Station 3 from Molecular Devices (San Jose, CA, USA), was employed to record the absorbance at a wavelength of 490 nm, which was subsequently utilized to compute the relative cell viability.

### 2.7. LDH Release

Adherence to the manufacturer’s guidelines was maintained during the employment of the LDH cytotoxicity assay kit. Post the aforementioned treatment, a volume of 80 μL of the supernatant was amalgamated with 60 μL of assay solution, followed by a period of incubation facilitated at ambient temperature for a duration of 30 min. Subsequent absorbance observations were conducted utilizing a microplate reader, with a specified wavelength of 490 nm. The quantification of relative LDH release was expressed as a percentage, representing the zenith of enzyme activity, accounting for both intracellular and extracellular LDH.

### 2.8. Intracellular Calcium Content

Intracellular calcium was detected using the fluorescent probe Fluo-3 AM (S1056, Beyotime Biotechnology, Shanghai, China). The cells were cultured by using Fluo-3AM (2.5 μM) at 37 °C for 60 min in the darkness for fluorescence probe loading and then washed properly to remove extracellular dye. The intensity exhibited by the fluorescence was recorded on the microplate reader under wavelength for emission reaching 530 nm, as well as the wavelength for excitation reaching 488 nm. It should be noted that this assay reflects relative changes in intracellular calcium-associated fluorescence intensity rather than absolute calcium concentrations.

### 2.9. Production of Intracellular ROS

The assessment of intracellular ROS levels was accomplished utilizing an ROS detection tool (Catalog No. S0033S, Beyotime Biotechnology). Subsequent to the treatment phase, cells were interfaced with a fluorescent probe through the introduction of 2′, 7′-dichlorofluorescein (DCFH-DA) solution, derived from the kit, at a concentration of 10 μM. Following a 30 min incubation period under dark conditions and at a regulated temperature of 37 °C, a triple wash was performed on the cells employing a serum-free medium. Utilizing a fluorescent microplate reader from Molecular Devices, San Jose, CA, USA, fluorescence intensity was calculated, operating at an excitation wavelength and an emission wavelength of 485 nm and 520 nm, respectively.

### 2.10. Measurement of Mitochondrial Membrane Potential (MMP)

PC12 cells were placed into six-well plates, attaining a density of 3.0 × 10^5^ cells per well, followed by a 24 h incubation period at a stable temperature of 37 °C. In the subsequent step, the cells underwent drug treatment maintaining the aforementioned conditions. They were then subjected to staining utilizing the fluorescent probe JC-1 for a duration of 20 min, maintained at 37 °C and shielded from light exposure (C2006, Beyotime Biotechnology). Afterward, a trio of washes was executed using the JC-1 washing solution, after which an equivalent number of cells were allocated into 96-well black plates. Following this, fluorescence intensity readings were captured using a fluorescent microplate reader, with the excitation wavelength defined at 488 nm and registering emission wavelengths at both 590 nm and 515 nm.

### 2.11. Flow Cytometric Analysis

PC12 cells, subjected to drug treatment, were strategically seeded into six-well culture dishes. Following a culture period spanning 24 h, the cells were meticulously harvested, subsequently washed, and carefully resuspended using phosphate-buffered saline (PBS). Thereafter, they were incubated, away from light exposure, with a mixture of 5 μL Annexin V-FITC and 5 μL of propidium iodide for a quarter of an hour (C1062, Beyotime Biotechnology, Shanghai, China). Utilizing flow cytometry (BD Biosciences, San Jose, CA, USA), the rate of apoptosis within the cell population was quantitatively assessed.

### 2.12. Proteomics Analysis

#### 2.12.1. Sample Preparation

PC12 cells were divided into three groups: the control group (untreated cells cultured under normal conditions), the model group (cells treated with 500 µM MPP^+^), and the drug group (cells treated with 500 µM MPP^+^ combined with 25, 50, 100 μM PF). At 48 h post-seeding, the culture medium was replaced with serum-free medium containing the respective treatments. After an additional 48 h of incubation, the cells were collected for analysis. Samples from each group were subjected to sonication in lysis buffer on ice three times using a high-intensity ultrasonic processor. The lysates were then centrifuged at 12,000× *g* for 10 min at 4 °C to remove debris. The resulting supernatants were collected, and protein concentrations were determined using a BCA kit (Thermo Fisher Scientific, Waltham, MA, USA) according to the manufacturer’s instructions.

#### 2.12.2. LC-MS/MS Analysis

Thermo Q-Exactive-Plus MS system (Waltham, MA, USA) was used to analyze the peptides. The mobile phase was 0.1% (*v*/*v*) formic acid (A) and acetonitrile (B) with linear elution (0.4 μL/min) from 4% B to 35% B over 90 min. The mass spectrometry data were compared to those of the UniProt protein database. Proteins were quantified using MaxQuant software (version 1.6.10.43).

#### 2.12.3. Bioinformatic Analysis

GO analysis was performed using the DAVID database, and the top 10 enriched terms were visualized. KEGG pathway analysis of the proteomics data was conducted using ClueGO (version 2.5.9) and KOBAS 3.0, respectively, while topological analysis was performed with the CytoNCA plug-in (version 2.1.6).

The present study did not generate a new TMT proteomic dataset; instead, the previously reported dataset [12] was reanalyzed specifically to investigate pathways potentially associated with the neuroprotective effects of PF.

### 2.13. Western Blotting

Western blot was performed to detect the expression of β-Actin, t-PI3K, p-PI3K, t-Akt, p-Akt, Bcl-2, Bax, cleaved-Caspase-3, t-ERK1/2, p-ERK1/2, t-p90RSK, p-p90RSK, t-CREB, p-CREB, BDNF, in PC12 cells. Extracted proteins were separated by 15% sodium dodecyl sulfate polyacrylamide gel electrophoresis (SDS-PAGE) and then transferred to polyvinylidene fluoride (PVDF) membrane. After closure with skimmed milk at room temperature, the membranes were incubated with β-Actin (1:10,000), t-PI3K (1:4000), p-PI3K (1:2000), t-Akt (1:10,000), p-Akt (1:1000), Bcl-2 (1:1000), Bax (1:2000), cleaved-Caspase-3 (1:1000), t-ERK1/2 (1:3000), p-ERK1/2 (1:3000), t-p90RSK (1:1000), p-p90RSK (1:1000), t-CREB(1:1000), p-CREB (1:1000), BDNF (1:1000). The primary antibodies underwent incubation at a temperature of 4 °C through an overnight period, which was subsequently followed by an additional re-incubation phase involving horseradish peroxidase-linked secondary antibody. The concluding steps involved the identification and quantification of protein bands present on the membranes, which was accomplished employing ECL Western blotting detection reagents and quantified utilizing Image J software (version 1.54f), sourced from the National Institutes of Health, located in Bethesda, MD, USA.

### 2.14. Validation of PF Effects on BDNF/ERK1/2/p90RSK and PI3K/Akt/CREB Pathways in an MPP^+^-Induced PC12 Cell Model

The PC12 cells were systematically categorized into five distinct groups: control, model, MPP^+^ + PF (where PC12 cells encountered treatment with 100 μM PF), MPP^+^ + PF + LY294002, and MPP^+^ + PF + PD98059 groups. Initially, the cells experienced a 30 min incubation period with either LY294002 (at a concentration of 25 μM) or PD98059 (concentration being 20 μM), following which they were incubated with either DMEM or PF at a concentration of 100 μM, sustained for a duration of 24 h. Subsequent to these treatments, the cells were subjected to injury induced by MPP^+^.

### 2.15. ERK1/2 Silencing by siRNA

ERK1/2 siRNA, utilized in this experiment, was acquired from SantaCruz Biotechnology (Dallas, TX, USA), through Comate Bioscience Co., Ltd (Changchun, China). The genetic sequence corresponding to ERK1 was documented as 5′-GGCCUCAAGUACAUACACUTT AGUGUAUGUACUUGAGGCCTT-3′, whereas ERK2 presented the sequence 5′-CCUGAGAGGAUUAAAGUAUTT AUACUUUAAUCCUCUCAGGTT-3′. PC12 cells, for the experiment, were cultured utilizing DMEM medium, which was enriched with 10% FBS and inclusive of antibiotics, maintaining this environment for a duration of 24 h. This was followed by a transfection process, leveraging Lipofectamine 2000 for a temporal span of 6 h, employing the specifically synthesized ERK1/2 siRNA. Subsequent to this, the medium was transitioned to DMEM containing 10% serum, and this condition was sustained for an additional 48 h, after which the following experiments were conducted.

### 2.16. Statistical Analysis

Data were presented as mean ± standard error mean (S.E.M.) in the Results section. The analytical evaluation of the attained data was conducted utilizing GraphPad Prism software, sourced from Graph-Pad Software Inc., located in San Diego, CA, USA (GraphPad Prism 4, Graph Pad Software, Inc., San Diego, CA, USA). Within the context of this research endeavor, the application of ANOVA was chosen for data analysis, which was subsequently complemented by the Tukey test, applied specifically for conducting multiple comparison assessments. A *p*-value less than 0.05 was interpreted as statistically significant, while a *p*-value below 0.01 was deemed to illustrate a high level of statistical significance.

## 3. Results

### 3.1. Paeoniflorin Alleviates Motor Dysfunction in MPTP-Induced Parkinson’s Disease Model Mice

**Figure 2:**
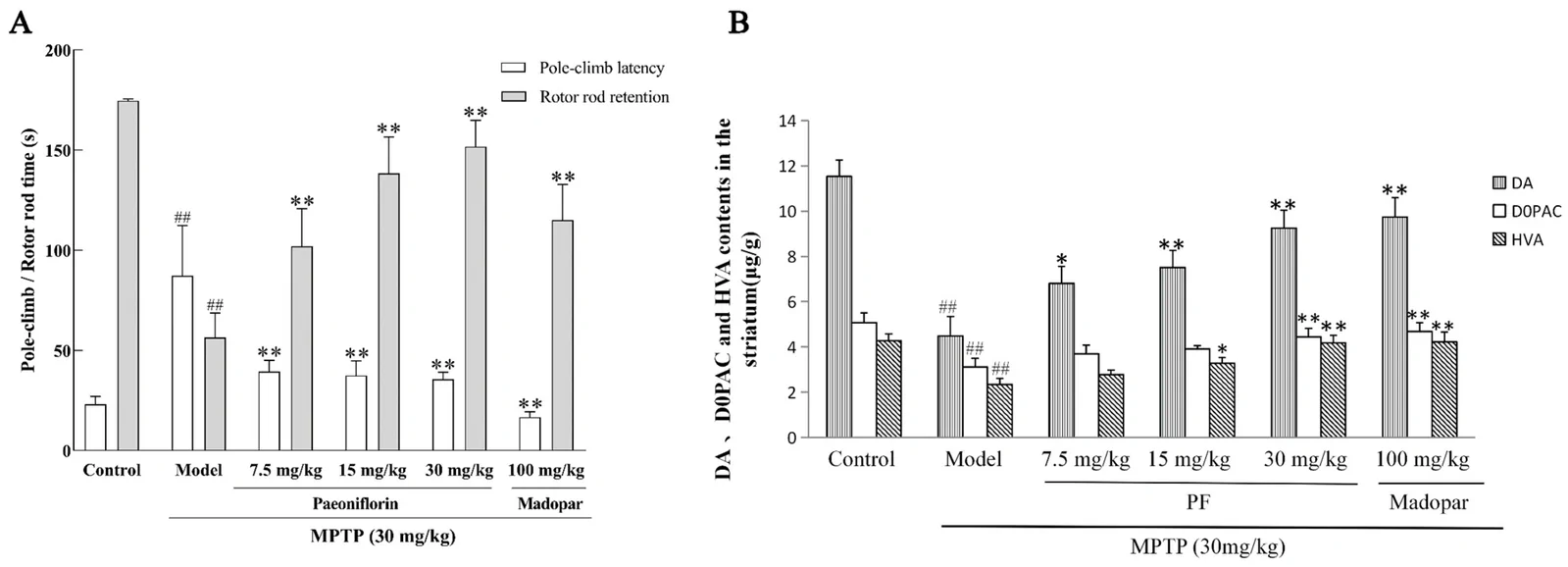
Effects of paeoniflorin (PF) on behavioral performance and striatal dopamine metabolism in MPTP-induced Parkinson’s disease model mice. (**A**) Behavioral assessment: pole-climb latency and rotarod retention time. White bars represent pole-climb latency, gray bars represent rotarod retention time. (**B**) Striatal DA, DOPAC and HVA contents reflecting dopamine metabolism. Mice were treated with MPTP (30 mg/kg) in the presence or absence of PF (7.5, 15, 30 mg/kg; low-, medium-, high-dose groups) or Madopar (100 mg/kg, positive drug group). Data are mean ± SEM (*n* = 12 per group per biological replicate; three independent biological replicates). ## *p* < 0.01 vs. control group; * *p* < 0.05, ** *p* < 0.01 vs. MPTP model group.

To investigate the ameliorative effect of paeoniflorin on motor dysfunction in MPTP-induced Parkinson’s disease model mice, pole-climb and rotarod tests were performed to evaluate the motor coordination of mice in each group. Mice in the control group exhibited short pole-climb latency and long rotarod retention time, indicating intact motor function. After MPTP administration, model group mice showed markedly prolonged pole-climb latency and shortened rotarod retention time, with extremely significant differences compared with the control group (## *p* < 0.01), which suggested obvious bradykinesia and impaired balance coordination in model mice. Compared with the model group, mice treated with 7.5, 15, 30 mg/kg paeoniflorin displayed significantly reduced pole-climb latency and extended rotarod retention time (** *p* < 0.01), and the therapeutic effect was elevated with an increase in paeoniflorin dose. Intervention with positive drug Madopar also significantly recovered the two behavioral indicators of mice (** *p* < 0.01) (Figure 2A). These results demonstrate that paeoniflorin can effectively reverse MPTP-induced motor impairment and alleviate bradykinesia and balance disorder in Parkinson’s disease mice.

### 3.2. PF Alters Striatal DA and Dopamine Metabolite Levels in MPTP-Treated Mice

As shown in Figure 2B, MPTP administration significantly reduced the levels of striatal dopamine (DA), 3,4-dihydroxyphenylacetic acid (DOPAC), and homovanillic acid (HVA) compared with the control group. PF treatment markedly reversed these alterations, with the highest-dose PF (30 mg/kg) and the positive-control Madopar (100 mg/kg) showing a stronger restorative effect on DA and its metabolites (*p* < 0.01). Madopar treatment also significantly improved the levels of DA, DOPAC, and HVA. These results indicate that PF effectively alleviates MPTP-induced dopaminergic dysfunction by restoring striatal neurotransmitter levels.

### 3.3. PF Prevents the Apoptosis Caused by MPP^+^

Based on our previous research findings that confirmed the neuroprotective efficacy of paeoniflorin (PF) in neurological injury models, the present study further explored the underlying molecular mechanism of PF against MPP^+^-induced PC12 cell damage. According to the validated safe and effective concentration range of PF reported in our prior study, 25, 50 and 100 μmol/L PF were selected for the in vitro mechanistic experiments in this work [12].

**Figure 3:**
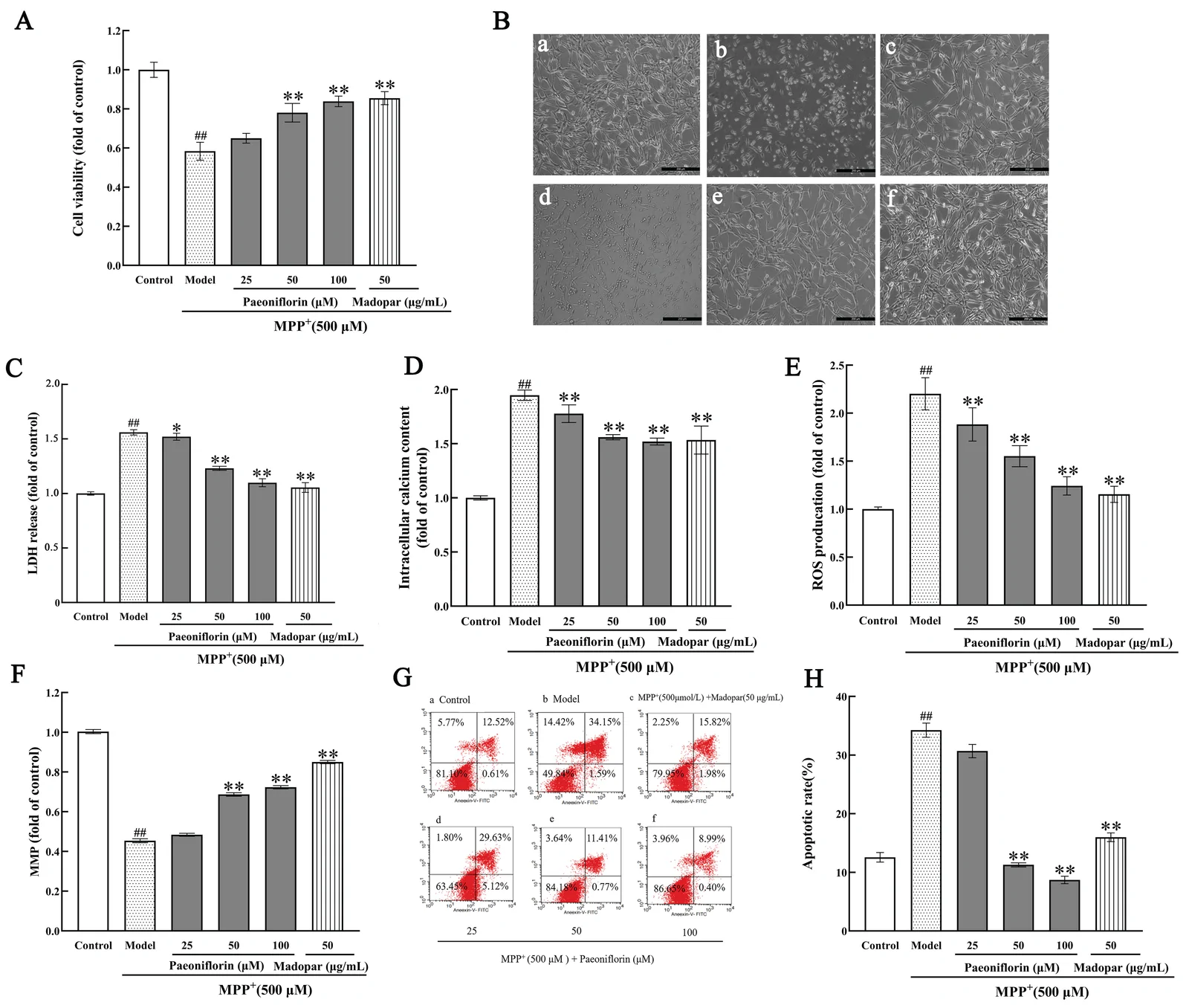
Paeoniflorin (PF) alleviates MPP^+^-triggered cytotoxicity, mitochondrial dysfunction and apoptosis in PC12 cells. (**A**) MTT assay showing the cytoprotective effects of PF (25, 50 and 100 μM) against MPP^+^-induced damage. (**B**) Representative phase-contrast images of PC12 cell morphology: (**a**) control, (**b**) MPP^+^-exposed Model group, (**c**) MPP^+^ + Madopar (50 μg/mL), (**d**) MPP^+^ + PF (25 μM), (**e**) MPP^+^ + PF (50 μM), (**f**) MPP^+^ + PF (100 μM). Scale bar = 200 μm. (**C**) LDH release. (**D**) Intracellular calcium content. (**E**) Intracellular ROS levels. (**F**) Mitochondrial membrane potential (MMP). (**G**) Representative flow cytometry plots of apoptotic cells. (**H**) Quantification of apoptotic rate. PC12 cells were treated with MPP^+^ (500 μM) in the presence or absence of PF (25, 50, 100 μM) or Madopar (50 μg/mL). Data are presented as mean ± SEM. *n* = 6 biological replicates for all assays. ## *p* < 0.01 vs. control group; * *p* < 0.05, ** *p* < 0.01 vs. MPP^+^-treated model group.

As shown in Figure 3A, MPP^+^ exposure significantly decreased the viability of PC12 cells compared with the control group (*p* < 0.01). Treatment with 50 μM PF, 100 μM PF or 50 μg/mL Madopar remarkably restored cell survival relative to the MPP^+^-injured model group (*p* < 0.01, *p* < 0.01, *p* < 0.01).

Microscopic observation of cell morphology (Figure 3B) revealed distinct morphological features in each group. PC12 cells in the control group displayed intact cell bodies and clear cell boundaries. After MPP^+^ intervention, the number of viable cells decreased, accompanied by cell body shrinkage and pyknosis. Treatment with PF or Madopar increased cellular interconnections and effectively reversed MPP^+^-induced morphological damage in PC12 cells.

### 3.4. PF Suppresses the Release of LDH, Increase in Ca^2+^ Overload and ROS Levels in the MPP^+^-Induced Cells

Upon contrasting with the control group, treating PC12 cells with 500 µM MPP^+^ for a duration of 24 h conspicuously facilitated the release of LDH (*p* < 0.01) and amplified Ca^2+^ concentration (*p* < 0.01), in addition to markedly elevating the levels of intracellular ROS (*p* < 0.01). Conversely, when PF (25, 50, and 100 μmol/L) was utilized as a pre-protective agent for a span of 48 h prior to introducing MPP^+^ injury, there was a notable diminution in LDH release (*p* < 0.05, *p* < 0.01, *p* < 0.01), Ca^2+^ concentration (*p* < 0.05, *p* < 0.01, *p* < 0.01), and intracellular ROS levels (*p* < 0.01, *p* < 0.01, *p* < 0.01), illustrated in Figure 3C–E.

### 3.5. PF Alleviates MPP^+^-Induced Mitochondrial Dysfunction and Apoptosis in PC12 Cells

Compared with the control group, the mitochondrial membrane potential of PC12 cells in the MPP^+^ group was significantly reduced (*p* < 0.01), while pretreatment with 50 μM and 100 μM PF as well as Madopar markedly elevated the mitochondrial membrane potential of MPP^+^-treated cells (all *p* < 0.01), suggesting that MPP^+^ impairs PC12 cells by decreasing mitochondrial membrane potential, and PF can counteract this MPP^+^-induced damage by enhancing mitochondrial membrane potential (Figure 3F).

In addition, flow cytometry combined with AnnexMPPin V-FITC/PI double staining was used to detect the effect of PF on MPP^+^-induced apoptosis (Figure 3G), and the results showed that MPP^+^ significantly induced the apoptosis of PC12 cells compared with the control group (*p* < 0.01). Pretreatment with 50 μM PF, 100 μM PF and Madopar exerted significant alleviating effects on MPP^+^-induced apoptosis of PC12 cells (all *p* < 0.01, Figure 3H).

### 3.6. Proteomics Identified the Protein and Pathways Related to the Effect of PF Against PD

To further explore the molecular mechanisms underlying the neuroprotective effects of paeoniflorin (PF), we performed a pathway-focused reanalysis of the TMT proteomic dataset generated in our previous study [12]. The dataset comprised 6405 identified proteins, of which 5525 were quantitatively measured. As reported previously [12], multiple-testing correction was applied to the statistical analyses, and the identified differentially expressed proteins (DEPs) were further evaluated using false discovery rate (FDR)-adjusted *p* values.

**Figure 4:**
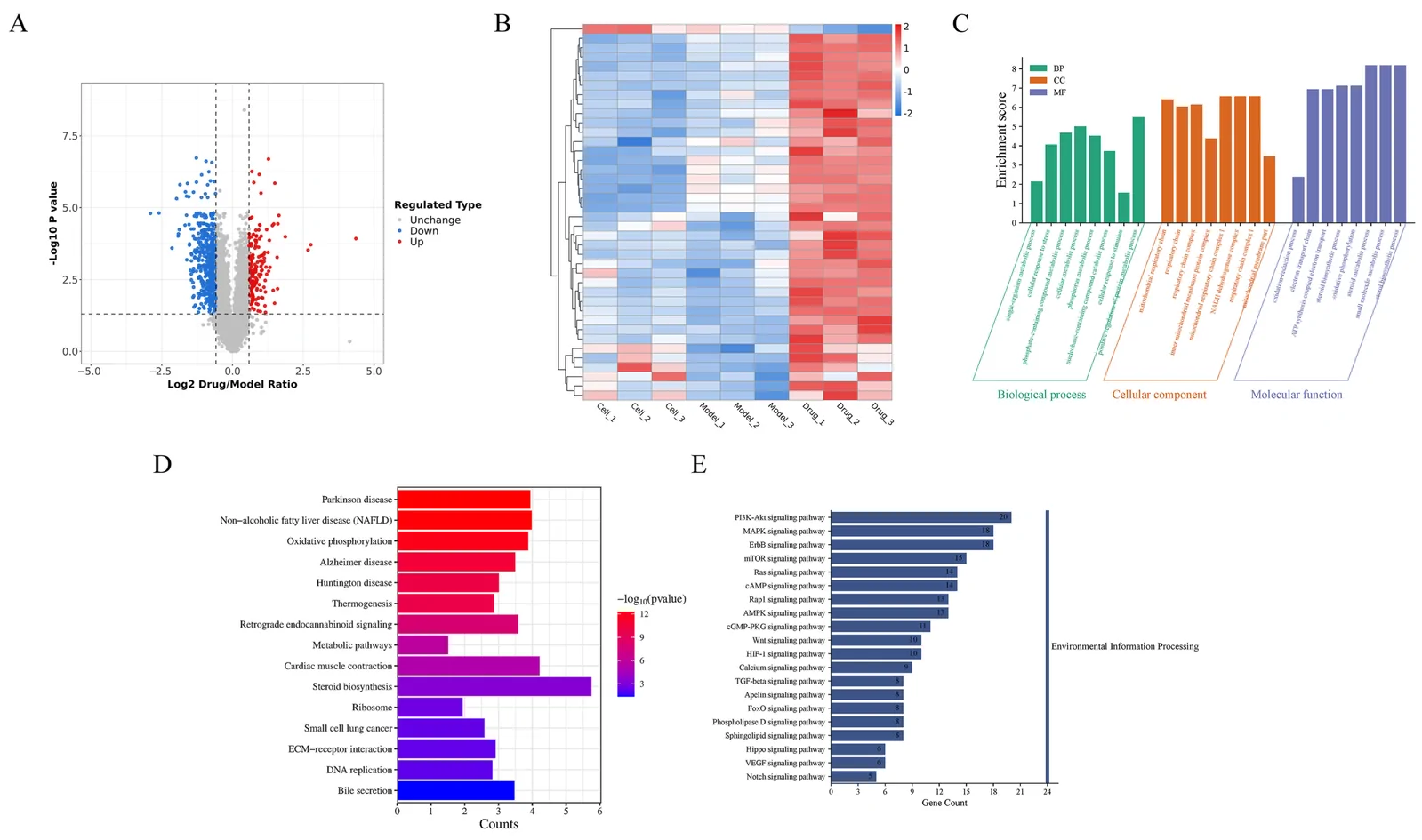
Proteomic profiling and bioinformatics analysis of PF-regulated proteins in MPP^+^-treated PC12 cells. (**A**) Volcano plot showing DEPs between PF-treated and MPP^+^ groups. Dotted lines represent the screening thresholds for log_2_(fold change) and P value. (**B**) Heatmap of hierarchical clustering of 47 DEPs across ctrl, model, and PF groups. (**C**) GO enrichment analysis of DEPs (BP, biological process; CC, cellular component; MF, molecular function). (**D**) Disease enrichment analysis highlighting Parkinson’s disease-related pathways. (**E**) KEGG pathway enrichment analysis of DEPs under Environmental Information Processing.

In the original proteomic analysis, an initial screening based on a fold change (FC) ≥1.5 or ≤0.67 and a nominal *p* value < 0.05 identified 282 proteins that differed between the model and cell groups, including 190 upregulated and 92 downregulated proteins. Similarly, 613 proteins were identified as differentially expressed between the drug and model groups, including 256 upregulated and 357 downregulated proteins. Because nominal *p* values alone may be insufficient for large-scale proteomic datasets, a more stringent FDR-controlled analysis was further considered. Using an FDR-adjusted *p* value (PFDR) < 0.05, 41 DEPs were identified in the model–cell comparison, including 29 upregulated (red) and 12 (blue) downregulated proteins, whereas 47 DEPs were identified in the drug–model comparison, including 14 upregulated and 33 downregulated proteins (Figure 4A). Thus, the FDR-controlled DEP sets were used as the principal basis for the subsequent pathway-focused interpretation.

The cluster heatmap illustrated the expression patterns of the 47 FDR-controlled DEPs identified in the drug–model comparison, including 14 (red) upregulated and 33 (blue) downregulated proteins (Figure 4B). Gene Ontology (GO) analysis categorized these DEPs into biological process (BP), cellular component (CC), and molecular function (MF) terms. The enriched terms were mainly associated with mitochondrial respiratory chain activity, cell membrane components, and small-molecule binding (Figure 4C), suggesting that PF treatment may influence cellular energy metabolism and stress-related processes in MPP+-injured cells.

KEGG pathway enrichment analysis identified 78 significantly enriched pathways. Among these, several pathways were closely associated with neurodegeneration and cellular stress responses, including Parkinson’s disease, oxidative phosphorylation, Alzheimer’s disease, Huntington’s disease, and ECM–receptor interaction (Figure 4D). The enriched pathways also included the PI3K-Akt, MAPK, ErbB, mTOR, Ras, and cAMP signaling pathways, indicating that multiple signaling networks may contribute to the cellular response to PF treatment. Notably, oxidative phosphorylation was connected with several neurodegenerative and metabolic pathways through shared protein involvement, further suggesting a potential relationship between mitochondrial function and PF-mediated neuroprotection.

To further prioritize candidate mechanisms for experimental validation, we integrated the GO and KEGG enrichment results with the functional relationships among the FDR-controlled DEPs. This analysis highlighted the PI3K-Akt and MAPK-related signaling networks as particularly relevant to neuronal survival, oxidative stress, and apoptosis. Detailed examination of the PI3K-Akt pathway further indicated a prominent association of BDNF with this signaling network. Among the identified proteins associated with this pathway, BDNF, CREB, PI3K, and Akt were, therefore, prioritized as candidate molecular components potentially involved in PF-mediated neuroprotection.

**Figure 5:**
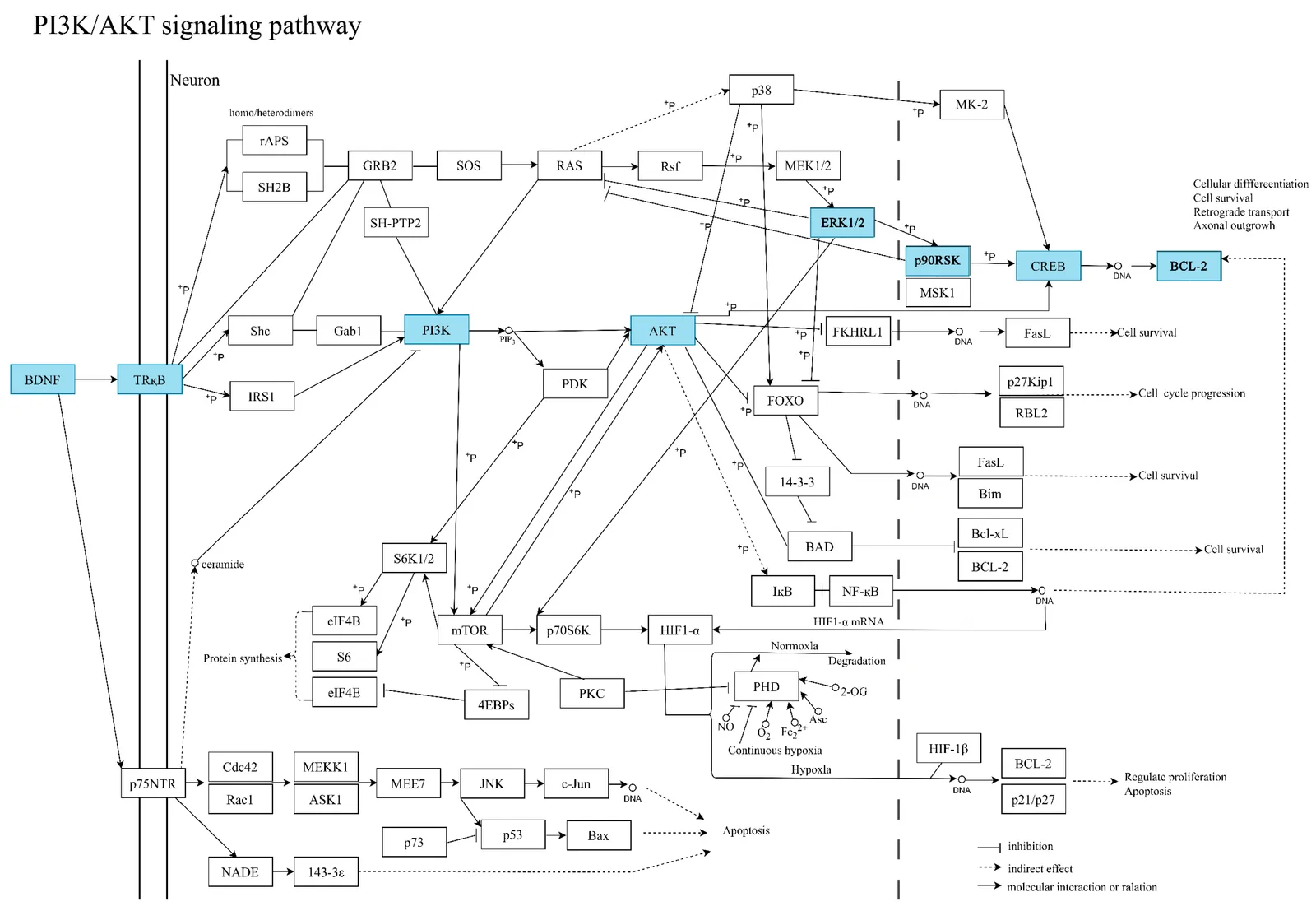
Integrated signaling network associated with the neuroprotective effects of PF based on proteomic and pathway analyses. The network was constructed by integrating the previously generated TMT proteomic dataset with KEGG pathway enrichment and functional analysis. The network illustrates the potential relationships among PF-associated signaling pathways and downstream molecular targets. Molecules highlighted with a distinct blue background indicate key targets experimentally examined in the present study, including ERK1/2, p90RSK, BCL-2, BAX, and other related proteins. Other molecules represent candidate components identified through pathway integration and are included to provide the broader mechanistic context. “+p” indicates phosphorylation-mediated activation; “→” indicates molecular interaction or regulatory relationship; “⊥” indicates inhibitory effect; dashed arrows indicate indirect regulatory effects.

Importantly, the selection of these candidate pathways and proteins was based on the proteomic and pathway enrichment results rather than solely on previously reported mechanisms. The proteomic dataset, therefore, served as a discovery-based starting point for identifying candidate signaling mechanisms, while the present study focused on pathway-level interpretation and subsequent experimental validation. Based on the integrated analysis, the PI3K/Akt and ERK/CREB signaling pathways were selected for further investigation because of their potential relevance to cell survival and neuroprotective responses. An integrated signaling network linking the proteomic findings with the candidate pathways and experimentally investigated molecular targets was subsequently constructed (Figure 5). This network provides a mechanistic framework connecting the discovery-based proteomic analysis with the molecular mechanisms examined in the subsequent experiments.

### 3.7. PF Regulates Apoptosis-Related Protein Expression and Activates the PI3K/Akt Signaling Pathway in PC12 Cells

**Figure 6:**
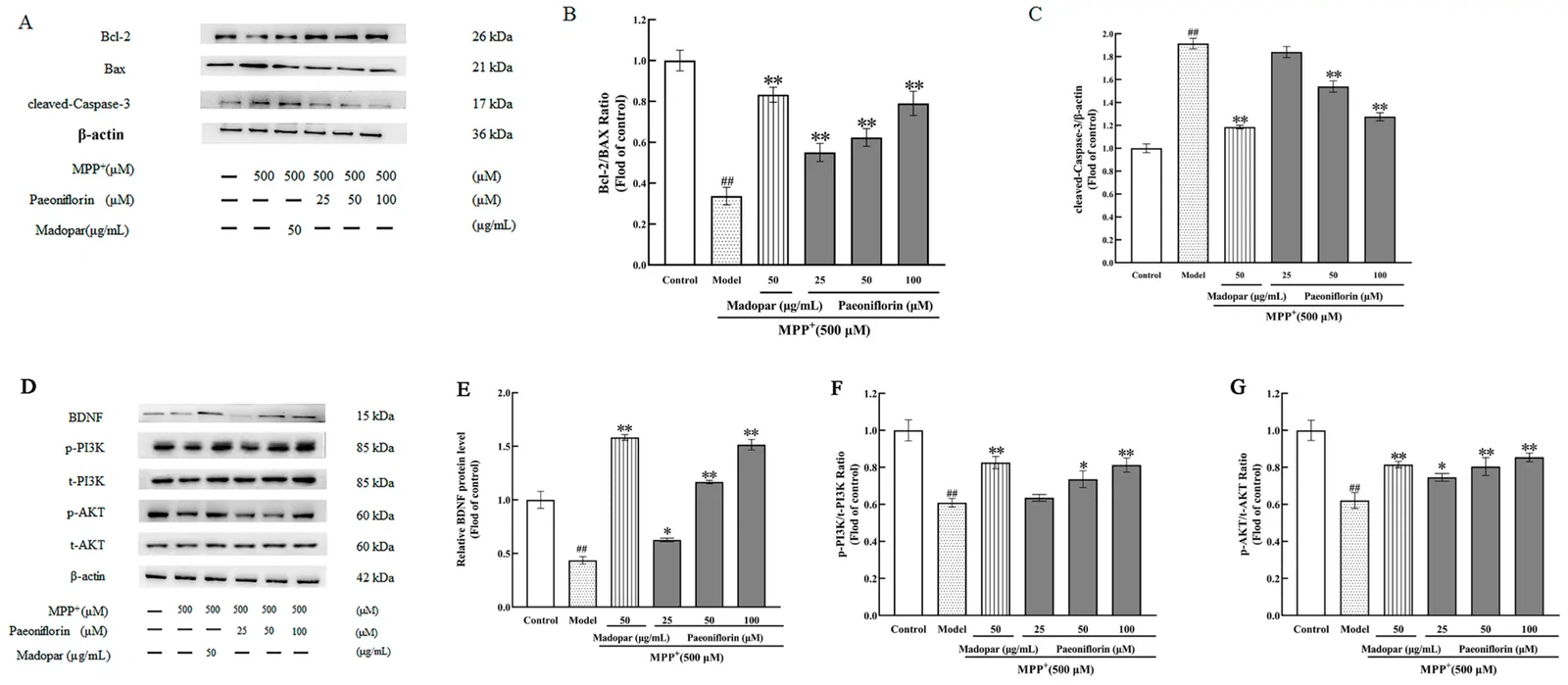
PF regulates apoptosis-related proteins and the BDNF/PI3K/AKT pathway in MPP^+^-treated PC12 cells. (**A**,**D**) Representative Western blots. (**B**) Bcl-2/Bax ratio. (**C**) Cleaved-Caspase-3/β-actin ratio. (**E**) BDNF level. (**F**) p-PI3K/t-PI3K ratio. (**G**) p-AKT/t-AKT ratio. PC12 cells were treated with MPP^+^ (500 μM) in the presence or absence of PF (25, 50, 100 μM) or Madopar (50 μg/mL). Data are mean ± SEM (*n* = 3 biological replicates). ## *p* < 0.01 vs. Control; * *p* < 0.05, ** *p* < 0.01 vs. MPP^+^ model group.

To explore the protective mechanism of PF against MPP^+^-induced apoptosis, Western blot analysis was performed to detect the expression levels of apoptosis-related proteins Bcl-2/Bax and cleaved-Caspase-3 in PC12 cells (Figure 6A). The results showed that MPP^+^ treatment led to a significant downregulation in the Bcl-2/Bax ratio and a notable upregulation in cleaved-Caspase-3 protein expression compared with the control group (both *p* < 0.01). Pretreatment with PF at concentrations of 25, 50 and 100 μM markedly inhibited the MPP^+^-induced decrease in the Bcl-2/Bax ratio (all *p* < 0.01), and PF at 50 and 100 μM also significantly attenuated the MPP^+^-triggered cleavage of Caspase-3 (both *p* < 0.01), demonstrating that PF alleviates MPP^+^-induced apoptosis in PC12 cells by regulating the expression of key apoptosis-related proteins (Figure 6B,C). Given that the PI3K/Akt signaling cascade is implicated in the mitigation of neuronal apoptosis in Parkinson’s disease murine models, the expression levels of BDNF and PI3K/Akt pathway-related proteins (p-PI3K, t-PI3K, p-AKT, t-AKT) were further detected. MPP^+^ treatment resulted in a significant reduction in the protein levels of BDNF, p-PI3K and p-AKT in PC12 cells, accompanied by a pronounced decrease in the BDNF, p-PI3K/t-PI3K and p-AKT/t-AKT ratios relative to the control group (all *p* < 0.01, Figure 6D). Notably, PF intervention at 25, 50, 100 μM significantly reversed the MPP^+^-induced downregulation of BDNF expression (*p* < 0.05, *p* < 0.01, *p* < 0.01), while PF at 50 and 100 μM effectively counteracted the MPP^+^-caused reduction in p-PI3K/t-PI3K (*p* < 0.05, *p* < 0.01) and p-AKT/t-AKT ratios (both *p* < 0.01) in a dose-responsive manner (Figure 6E–G), indicating that PF can activate the PI3K/Akt signaling pathway by upregulating BDNF protein expression.

### 3.8. PF Exerts Neuroprotective Effects Against MPP^+^-Induced Injury via BDNF/PI3K/AKT and ERK1/2/p90RSK/CREB Signaling Pathways

**Figure 7:**
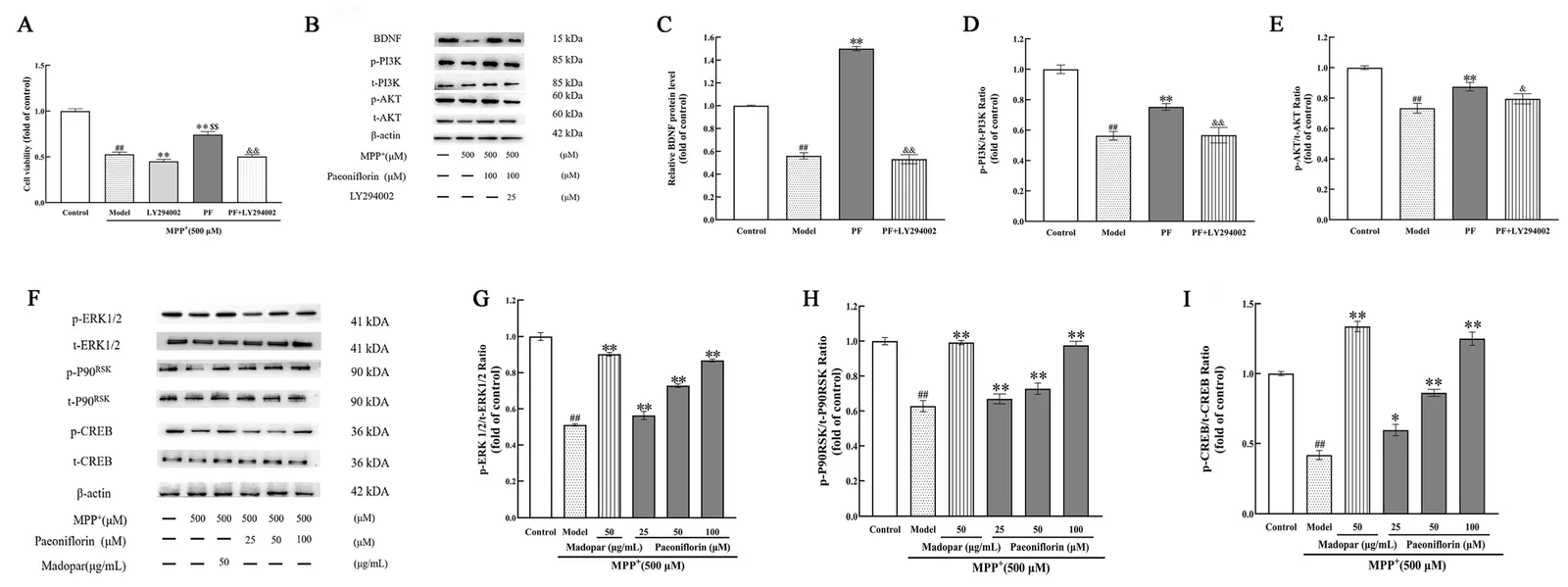
PF activates the PI3K/AKT-ERK1/2-p90RSK-CREB signaling pathway against MPP^+^-induced cell injury. (**A**) Cell viability of PC12 cells co-treated with the PI3K inhibitor LY294002. (**B**,**F**) Representative Western-blot bands. (**C**–**E**,**G**–**I**) Densitometric analysis of (**C**) BDNF protein level, (**D**) p-PI3K/t-PI3K ratio, (**E**) p-AKT/t-AKT ratio, (**G**) p-ERK1/2/t-ERK1/2 ratio, (**H**) p-p90RSK/t-p90RSK ratio, and (**I**) p-CREB/t-CREB ratio. PC12 cells were treated with MPP^+^ (500 μM) in the presence or absence of PF (25, 50, 100 μM), Madopar (50 μg/mL), or the PI3K inhibitor LY294002 (25 μM). Data are mean ± SEM (*n* = 3 biological replicates). ## *p* < 0.01 vs. control; ** *p* < 0.01 vs. MPP^+^ model group; $$ *p* < 0.01 vs. corresponding LY294002 inhibitor group; && *p* < 0.01 vs. PF-treated group. * *p* < 0.01.

To verify that the neuroprotective effect of PF is mediated by the PI3K/AKT signaling pathway, LY294002 was used to inhibit PI3K activity. As illustrated in Figure 7A, the LY294002 group exhibited a markedly reduced cell viability under MPP^+^-induced injury (** *p* < 0.01 versus model group). The cell viability in the PF + LY294002 group was significantly higher than that in the LY294002-only group ($$ *p* < 0.01), whereas the PF group demonstrated significantly higher viability compared with both the LY294002 group and the PF + LY294002 group (** *p* < 0.01 and && *p* < 0.01, respectively). These findings indicate that LY294002 further aggravated MPP^+^-triggered cell damage and partially diminished the cytoprotective effect of PF, while PF still retained partial protective capacity even upon PI3K blockade.

Western blotting was further performed to detect the expression levels of BDNF, p-PI3K, t-PI3K, p-AKT and t-AKT proteins (Figure 7B–E). Compared with the model group, the MPP^+^ + PF + LY294002 combined treatment group showed no significant changes in BDNF expression, p-PI3K/t-PI3K and p-AKT/t-AKT ratios (all *p* > 0.05). By contrast, the expression of BDNF and the ratios of p-PI3K/t-PI3K and p-AKT/t-AKT were significantly decreased in the MPP^+^ + PF + LY294002 group relative to the PF intervention group (all *p* < 0.01). These results indicated that PF can activate the PI3K/Akt signaling pathway by upregulating BDNF protein expression, and LY294002 can reverse these regulatory effects, highlighting the crucial role of the PI3K/AKT signaling pathway in mediating the neuroprotective effect of PF.

In addition, the involvement of the ERK1/2/p90RSK/CREB signaling pathway in the neuroprotective effect of PF was also investigated. MPP^+^ treatment had no significant effect on the total protein expression of ERK1/2, p90RSK and CREB but significantly reduced the phosphorylation levels of p-ERK, p-p90RSK and p-CREB, leading to a marked decrease in p-ERK1/2/t-ERK1/2, p-p90RSK/t-p90RSK and p-CREB/t-CREB ratios (all *p* < 0.01). Pretreatment with PF significantly upregulated the expression levels of p-ERK, p-p90RSK and p-CREB while keeping the total protein levels of ERK, p90RSK and CREB stable, thus significantly increasing the ratios of p-ERK1/2/t-ERK1/2, p-p90RSK/t-p90RSK and p-CREB/t-CREB (all *p* < 0.05 or *p* < 0.01) (Figure 7F–I), demonstrating that the ERK1/2/p90RSK/CREB signaling pathway is also involved in the neuroprotective effect of PF against MPP^+^-induced cell injury.

### 3.9. PD98059 and ERK1/2 siRNA Restricts the ERK1/2/ p90RSK/CREB Signaling Pathway and Attenuates the Protective Effect of PF

**Figure 8:**
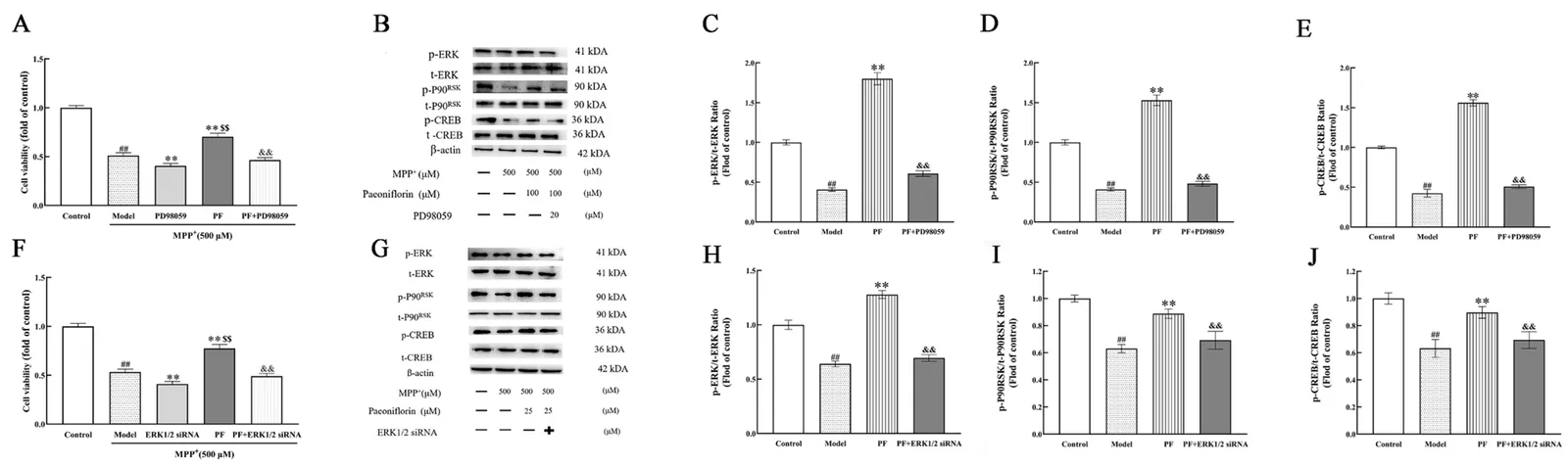
PF activates the ERK1/2-p90RSK-CREB signaling pathway to exert neuroprotective effects in MPP^+^-injured PC12 cells. (**A**) Cell viability of PC12 cells co-treated with the ERK1/2 inhibitor PD98059. (F) Cell viability of PC12 cells transfected with ERK1/2-specific siRNA. (**B**,**G**) Representative Western blot bands. (**C**–**E**,**H**–**J**) Densitometric quantification of (**C**) p-ERK/t-ERK ratio, (**D**) p-p90RSK/t-p90RSK ratio, (**E**) p-CREB/t-CREB ratio, (**H**) p-ERK/t-ERK ratio, (**I**) p-p90RSK/t-p90RSK ratio, and (**J**) p-CREB/t-CREB ratio. PC12 cells were treated with MPP^+^ (500 μM) in the presence or absence of PF, PD98059, or ERK1/2-specific siRNA. Both pharmacological inhibition and genetic knockdown of ERK1/2 abrogated PF-triggered pathway activation and neuroprotection. Data are expressed as mean ± SEM (*n* = 3 biological replicates). ## *p* < 0.01 vs. control; ** *p* < 0.01 vs. model; $$ *p* < 0.01 vs. corresponding inhibitor/siRNA group; && *p* < 0.01 vs. PF.

As illustrated in Figure 8A, the PD98059 group exhibited markedly reduced cell viability under MPP^+^-induced injury (** *p* < 0.01 versus model group). The cell viability in the PF + PD98059 group was significantly higher than that in the PD98059-only group ($$ *p* < 0.01), whereas the PF group demonstrated significantly higher viability compared with both the PD98059 group and the PF + PD98059 group (** *p* < 0.01 and && *p* < 0.01, respectively). These findings indicate that PD98059 further aggravated MPP^+^-triggered cell damage and partially diminished the cytoprotective effect of PF, while PF still retained partial protective capacity even upon ERK1/2 pharmacological inhibition.

Upon PD98059 intervention in the MPP^+^ + PF group, the elevations of p-ERK1/2/t-ERK1/2, p-p90RSK/t-p90RSK and p-CREB/t-CREB induced by PF were abolished. Compared with the model group, the MPP^+^ + PF + PD98059 group exhibited markedly decreased levels of p-ERK1/2/t-ERK1/2 (*p* < 0.01), p-p90RSK/t-p90RSK (*p* < 0.01), and p-CREB/t-CREB (*p* < 0.01).

As shown in Figure 8F, ERK1/2 siRNA transfection was associated with a significant reduction in cell viability following MPP^+^ stimulation compared with the model group (** *p* < 0.01). Co-treatment with PF partially improved cell viability compared with the ERK1/2 siRNA-only group ($$ *p* < 0.01). However, cell viability in the PF group remained significantly higher than that in both the ERK1/2 siRNA group and the PF + ERK1/2 siRNA group (** *p* < 0.01 and && *p* < 0.01, respectively). These findings suggest that interference with ERK1/2 signaling was associated with a reduction in the cytoprotective effect of PF, although part of the PF-mediated protection was retained following ERK1/2 siRNA treatment.

Consistent with these findings, ERK1/2 siRNA treatment reduced the PF-associated increases in the phosphorylation levels of ERK1/2, p90RSK, and CREB. Compared with the model group, the MPP^+^ + PF + ERK1/2 siRNA group showed significantly lower p-ERK1/2/t-ERK1/2, p-p90RSK/t-p90RSK, and p-CREB/t-CREB ratios (all *p* < 0.01). These results indicate that interference with ERK1/2 signaling was accompanied by attenuation of the PF-associated activation of the ERK1/2-p90RSK-CREB signaling cascade.

Taken together, the pharmacological inhibition and siRNA interference results pro-vide supportive evidence for the involvement of the ERK1/2-p90RSK-CREB signaling pathway in PF-mediated cytoprotection against MPP^+^-induced injury (Figure 8A–J). However, given that quantitative ERK1/2 knockdown efficiency and an appropriate scrambled-siRNA negative control were not included in the present study, these findings should be interpreted as supportive rather than definitive evidence of a causal role for ERK1/2 in PF-mediated neuroprotection.

## 4. Discussion

Parkinson’s disease (PD) is a common age-related degenerative disorder in the CNS characterized by behavioral abnormalities and motor dysfunction. The primary objective of PD treatment is to ameliorate clinical symptoms and decelerate the pathological progression. Within the realm of ethnomedicine, the long-standing use of accumulated medicinal herbs in clinical practice has bestowed a valuable resource for drug development, particularly for compounds exhibiting potent neuroprotective effects [11]. Previous studies have confirmed that PF, the main active component extracted from *Paeonia lactiflora* Pall, is capable of crossing the blood–brain barrier (BBB), albeit with limited permeability, and relevant in vitro experiments using MDCK-Mdr1 cells as a BBB model have elucidated its transport mechanism, which can be further regulated by components such as ligustilide, senkyunolide I and senkyunolide A to enhance its brain delivery by affecting the expression of P-glycoprotein (P-gp) [13]. It has been reported that PF administration can significantly improve cognitive impairment in animal models of neurodegenerative diseases; specifically, it can suppress neuronal ferroptosis to alleviate memory deficits in Alzheimer’s disease mice, which is closely related to the regulation of oxidative stress and neuroinflammation [26]. Moreover, PF can alleviate lipopolysaccharide (LPS)-induced neuroinflammation and depression-like behaviors through activating the Keap1/Nrf2/HO-1 signaling pathway, which also contributes to reducing brain tissue damage caused by neuroinflammation, along with inhibiting the TLR4/NF-κB pathway and reducing the release of pro-inflammatory cytokines (TNF-α, IL-1β, IL-6) [27]. These findings collectively indicate that PF, as a neuroprotective agent, has potential application value in the prevention and treatment of various neurodegenerative diseases, including Alzheimer’s disease, as well as related neuroinflammatory disorders.

Studies have shown that paeoniflorin can enhance the sensitivity of ER+ breast cancer cells to tamoxifen via the SIRT4/STAT3 pathway, serving as a potential candidate for combination therapy with tamoxifen in the treatment of ER+ breast cancer [28]. Additionally, paeoniflorin modulates microglia–astrocyte crosstalk through the HSP90AA1/HMGB1 pathway to alleviate neuropathic pain and inhibit inflammatory responses [29]. Extracts from the roots of *Paeonia lactiflora*, specifically total glycosides of *Paeonia lactiflora* (TGP), have demonstrated notable efficacy in addressing issues related to the central nervous system and disorders that involve neurodegeneration, with a particular emphasis on Parkinson’s disease [30,31,32]. Paeoniflorin (PF), identified as the predominant active constituent within *Paeonia lactiflora*, is perceived to contribute significantly to the plant’s neuroprotective properties [33,34,35]. Consequently, this study was devised to delve into the neuroprotective capacities of paeoniflorin, particularly within the context of Parkinson’s disease, and to explore the potential mechanisms underpinning its action.

As expected, repeated MPTP administration induced significant behavioral deficits, characterized by reduced spontaneous locomotion, prolonged descent latency in the pole-climb test and impaired motor coordination on the rotarod test, consistent with previous findings [36,37,38]. This study demonstrated that PF ameliorated MPTP-induced bradykinesia and motor dysfunction in mice (Figure 2A,B). Notably, while Madopar provided superior improvements in general locomotor activity, it exhibited weaker efficacy in improving motor coordination and bradykinesia. This distinction likely stems from their different mechanisms: Madopar acts via direct dopamine supplementation [39], whereas PF appears to exert its benefits primarily through neuroprotection of the nigrostriatal dopaminergic system. Consistent with this, PF treatment effectively attenuated MPTP-induced neurotoxicity and rescued dopaminergic neuronal death in the substantia nigra.

PC12, a cell line embodying neuroendocrine characteristics, displayed an elevated Caspase-3 and Bax/Bcl-2 ratio within the MPP^+^-treated PC12 cell model, indicating an escalation in apoptosis [40]. Consistent with our observations, a research article demonstrated that an enhancement in α-syn aggregation in dopaminergic neurons, a decline in Bcl-2 levels, and an impediment of autophagic flux in PD rats were instigated by ROT [41]. This phenomenon coincided with the amplified immunoreactivity of Caspase-3 in neurons, which stimulates the activity of cytoplasmic nucleic acid endonuclease, catalyzes the degradation of the cytoskeletal protein PARP, and orchestrates a plethora of morphological and biochemical transformations within apoptotic cells [42]. Previous demonstrations have established that neurotoxin exposure to neuronal cells induces cellular damage, prompting alterations in cell membrane permeability and, consequently, provoking a substantial surge in LDH release alongside intracellular Ca^2+^ overload [43,44]. The considerable influx of Ca^2+^ generates oxidative stress, a notable precipitator of apoptosis, due to its role in disrupting cytosolic calcium homeostasis and initiating the apoptotic program [45,46,47]. Initially, ROS are synthesized within the mitochondria, and when their concentration transcends a specific threshold, it induces a cascade of cellular impairments, damaging lipids, proteins, and DNA [48,49,50]. The disruption of mitochondrial function by ROS prompts a reduction in membrane potential, subsequently inducing apoptosis and furthering the progression of Parkinson’s disease [51,52]. This investigation unveiled that MPP^+^-induced cellular injury catalyzed an augmentation in LDH release, intracellular Ca^2+^ concentration, and reactive oxygen species levels, thus enhancing apoptosis. Nevertheless, the data also highlighted that PF possessed the capability to counteract these phenomena, demonstrating neuroprotective effects.

Proteomics has emerged as a critical tool for understanding disease progression and pharmacological mechanisms through the analysis of differentially expressed proteins (DEPs) in physiological or pathological settings. Different from the 282 DEPs obtained from MPTP model mice versus control mice in our previous animal study, the present cellular TMT-proteomic analysis identified 47 DEPs by comparing PF-treated PC12 cells against MPP^+^-injured PC12 cells. Tandem mass tag (TMT)-based proteomics provides a robust platform for relative quantification of large-scale protein expression across multiple samples [53,54]. Hence, we conducted TMT-based proteomics to investigate anti-PD mechanisms of PF. Totally, 5525 quantifiable proteins were determined according to proteomics analysis. Following PF intervention in comparison to the MPP^+^ group, we observed 47 DEPs, consisting of 14 upregulated proteins and 33 downregulated proteins, which are likely primarily located in dendrites, other parallel fibers to purkinje cell synapse and secretory granule. Moreover, KEGG pathway analysis of these DEPs demonstrates that the estrogen signaling pathway and calcium signaling pathway are closely connected with PF’s anti-PD potency.

BDNF, a crucial constituent of the nerve growth factor family, emerges as a secretory polypeptide pervasively located in the central nervous system and additional organs, acting dually in facilitating nerve regeneration and synaptic enlargement [55,56]. The BDNF-TrkB axis enhances synaptic expansion, nerve plasticity, connectivity; supports neuronal preservation; and thwarts apoptosis within the central nervous system [57]. The progression of Parkinson’s disease (PD) implicates the internal cellular signaling through the mitogen-activated protein kinase (MAPK) pathway [58]. ERK1/2, a prominent member of the MAPK family and cytoplasmic protein kinase, upon activation, swiftly traverses the nuclear membrane, modulating the functionality of particular transcription factors through phosphorylation. This action induces modifications in the expression or functionality of distinct proteins, thereby influencing cellular biological effects [59,60]. BDNF/TrkB-mediated ERK1/2 phosphorylation activates ribosomal S6 protein kinase (RSK) protein kinase substrates on the cellular membrane surface and within the cytoplasm and nucleus, facilitating their phosphorylation and concurrent nuclear entry to enhance the phosphorylation of pivotal transcription factors, such as CREBs [61]. Notably, RSK family members have been reported to play crucial regulatory roles in neuronal necroptosis, which is closely related to the pathological progression of PD [62,63]. This further supports the potential involvement of RSK in the neuroprotective mechanism mediated by PF in PD models. Phosphorylation of CREB, a pathway of noteworthy significance, enables transcription regulation by CREB and its phosphorylation, eventually guiding the gene expression of downstream factors like BDNF, influencing neuronal plasticity, neurotransmitter synthesis, and orchestrating activities related to synaptic plasticity such as neuronal cell proliferation and differentiation [64,65]. Moreover, the ERK phosphorylation degree serves as a pivotal indicator mirroring the intensity of BDNF/TrkB signaling activity. Research indicates CREB’s under-expression in a schizophrenia mouse model, while its overexpression exerts a negative regulation upon BDNF [66]. Further studies illustrate that activation of Raf/ERK1/2/p90RSK/CREB pathway proteins can maneuver ERK proteins by modulating CREB phosphorylation, subsequently directing the replication of target genes, including BDNF and Bcl-2 [59].

The PI3K/Akt pathway emerges as another pivotal conduit in the downstream signaling cascade of BDNF/TrkB, exemplifying a characteristic anti-apoptotic signaling route and assuming a substantial role in endorsing neuronal survival, synaptic plasticity, and antidepressant-analogous effects [67]. Inducing the activation of PI3K bestows cells with a crucial survival signal, enabling them to counteract apoptotic affronts [68]. Engaging the PI3K/Akt pathway enhances the phosphorylation degree of Akt, consequently triggering the cellular anti-apoptotic mechanisms. Such an augmentation in Akt phosphorylation not only bolsters the anti-apoptotic mechanisms within cells but also suppresses apoptosis, thereby solidifying Akt’s essentiality in preserving normal cerebral function [69]. By inhibiting the functionality of pro-apoptotic proteins and stalling apoptotic processes, Akt propels cellular survival [70]. Furthermore, Akt escalates the expression of Bcl-2, thereby fostering cellular survival. Such a Bcl-2 upregulation correlates with the activation of ERK1/2 and PI3K pathways, as well as increased levels of both activated phosphorylated RSK and phosphorylated CREB, serving as intersecting targets for ERK1/2 and PI3K pathways.

Moreover, Akt executes phosphorylation of BAD, a pro-apoptotic member of the Bcl-2 family, consequently mitigating the pro-apoptotic actions of BAD. Notably, Bcl-2 is incorporated within a protein family known for its apoptosis-inhibiting properties, and the elevation in Bcl-2 expression is recognized as a pivotal mechanism underpinning cellular survival [71,72]. The induction of oxidative stress can be ameliorated through the enhancement of Bcl-2 expression and the simultaneous diminution of Caspase-3 expression [73]. Research has illustrated that baicalin mitigates apoptotic neurotoxicity induced by ketamine in the neural development of rats [74]. It might be hypothesized that the ascension of Bcl-2 and the descent of Caspase-3 could act as downstream consequences of the PI3K/Akt and CREB/BDNF signaling pathways, a hypothesis that aligns with our observed findings. Furthermore, LY294002 exhibited an inhibitory impact on the PI3K/Akt pathway, which harmonizes with our observations. Experiments with MPP^+^-induced PC12 cells unveiled that PF could modulate the ERK1/2/p90RSK/CREB and BDNF/PI3K/Akt pathways, thereby imparting a protective shield to PC12 cells. Notably, the designated PI3K inhibitor LY294002 and the ERK inhibitor PD98059 could markedly counteract the influence of PF. Notably, BDNF acts as a key mediator of paeoniflorin-induced neuroprotection, yet we have not verified the direct causal relationship between upregulated BDNF and downstream pro-survival pathway activation as well as the final neuroprotective phenotype. Combining our TMT proteomic data and KEGG pathway enrichment analysis, we systematically mapped the downstream signaling network triggered by BDNF (Figure 5). After binding to its high-affinity receptor TrkB, BDNF governs neuronal survival and function via two core signaling axes:

PI3K/AKT pathway: Activated AKT transmits critical pro-survival signals to inhibit neuronal apoptosis. ERK1/2/p90RSK/CREB pathway: The BDNF/TrkB complex initiates the RAS/RAF/MEK cascade to activate ERK1/2, which further phosphorylates p90RSK and CREB to promote transcription of anti-apoptotic genes including Bcl-2.

Together, these two cascades form an integrated BDNF-dependent neuroprotective network, suggesting future work may focus on how paeoniflorin targets the PI3K/AKT and BDNF/TrkB/ERK1/2/p90RSK/CREB pathways.

Our data demonstrate that paeoniflorin synchronously activates both ERK1/2/p90RSK/CREB and PI3K/Akt signaling, boosts autocrine BDNF release, and consequently promotes neuronal survival and tissue repair. Although persistent artificial overactivation of these pleiotropic pathways may carry toxic risks, the pathway changes observed here are secondary pharmacological responses triggered by paeoniflorin treatment rather than forced direct activation. Accordingly, these findings provide a preliminary mechanistic framework and potential pharmacodynamic biomarkers, rather than definitive target validation. The correlation we observed between increased BDNF expression and enhanced pathway activity cannot confirm that PF’s protective actions are strictly BDNF-dependent. In the absence of TrkB inhibition, BDNF neutralization, or BDNF knockdown experiments in the current study, alternative upstream receptors or parallel signaling pathways cannot be excluded as independent triggers of PI3K and ERK activation. Therefore, the hypothesis that BDNF acts as a primary upstream trigger of these survival pathways remains preliminary and speculative. Further rigorous loss-of-function validations are required to clarify the exact dependency of PF-mediated neuroprotection on BDNF/TrkB signaling, which will be systematically implemented in our future mechanistic investigations.

It is necessary to acknowledge the inherent limitations of the PC12 cell model used in this study. PC12 cells derived from rat pheochromocytoma were selected for preliminary mechanistic screening due to their typical catecholaminergic characteristics, stable MPP^+^ responsiveness, and widespread acceptance in PD neurotoxicity research. Nevertheless, this cell line cannot fully recapitulate the biological properties of mature primary dopaminergic neurons in the mouse substantia nigra. All functional validations of the two core signaling pathways were performed in the in vitro PC12 cell system, whereas corresponding pathway inhibition and molecular verification in MPTP mouse brain tissues are lacking in the current work, which weakens the translational connection between in vitro signaling changes and in vivo neuroprotective outcomes. Although the published literature has confirmed the consistent regulatory trends of PF on PI3K/Akt and ERK/CREB pathways both in PC12 cells and mouse midbrain tissues, further in vivo intervention experiments are still required to solidify the in vivo regulatory functions of these pathways. Future studies will also prioritize primary dopaminergic neurons or human iPSC-derived neuronal models to improve the translational reliability of the mechanistic conclusions.

In addition, consistent with previous recognition, PF possesses relatively limited BBB permeability, which poses a potential challenge for its central pharmacological action [13]. Although multiple studies have verified that systemically administered PF can reach detectable therapeutic levels in brain tissue and directly regulate neuronal signaling, peripheral regulatory mechanisms, including gut microbiota remodeling and systemic peripheral anti-inflammatory responses [75], may also contribute indirectly to the overall neuroprotective effects. The relative contributions of central direct versus peripheral indirect mechanisms remain unclarified in the present study and will be comprehensively explored in our subsequent pharmacokinetic and microbiome-focused research.

Despite these pharmacokinetic limitations, PF remains a promising lead compound, as its neuroprotective activity and apparent ability to modulate neurotrophic and survival-related signaling provide a rationale for further pharmacological optimization. Strategies aimed at improving BBB permeability and CNS exposure [21] may facilitate the development of PF derivatives with enhanced therapeutic potential, while the modulation of PI3K/Akt and ERK/CREB signaling identified in the present study may provide a useful mechanistic framework for evaluating such optimized compounds. Nevertheless, several limitations of the present study should be acknowledged. Although our findings support the involvement of the BDNF/TrkB, PI3K/Akt, and ERK/CREB signaling pathways in the neuroprotective effects of PF, direct in vivo validation of these signaling events in brain tissue was not performed in the current study. Future studies should, therefore, focus on validating these signaling mechanisms in appropriate PD animal models, identifying the direct molecular target(s) of PF, and evaluating whether its protective effects can be reproduced in chronic or genetic models of PD. Further optimization of PF to improve BBB permeability, CNS exposure, and safety margins may also enhance its translational potential. Taken together, the current findings, together with previous in vivo evidence, support the potential neuroprotective effects of PF against MPTP/MPP^+^-induced dopaminergic neuronal injury and suggest that PF may represent a promising candidate for further investigation in the context of PD.

## 5. Conclusions

To sum up, this study identifies BDNF-associated PI3K/Akt and ERK/CREB signaling pathways as a key mechanism underlying the neuroprotective efficacy of PF against acute toxin-induced neurotoxicity in experimental PD models. In addition to demonstrating PF’s ability to alleviate MPTP-provoked behavioral deficits and protect dopaminergic neurons against toxin-mediated damage, we provide evidence suggesting an interaction between these two survival pathways that may contribute to PF’s neuroprotective effects. This novel mechanistic insight moves beyond a simple phenotypic description and elucidates how PF achieves its multifaceted protection against MPTP/MPP^+^-induced damage. Nevertheless, several important limitations of the present study should be explicitly addressed. First, all mechanistic explorations were performed in acute MPP^+^/MPTP toxin-induced PD models, which simulate abrupt neuronal injury rather than the slowly progressive and chronic pathological process of clinical PD. Notably, PF was predominantly applied as a pretreatment in our experimental settings; accordingly, our findings cannot support claims for therapeutic reversal of well-established PD-related pathology. Therefore, the efficacy and molecular mechanisms of PF in chronic PD models and genetic PD models require further verification, including post-injury therapeutic intervention studies. Second, although we observed PF-induced BDNF upregulation accompanied by pro-survival pathway activation, BDNF may serve as an important regulator correlated with paeoniflorin-mediated neuroprotection; however, causal dependency on BDNF/TrkB signaling remains unproven, since direct loss-of-function inhibition or knockdown of BDNF/TrkB was not performed in the current study, and alternative parallel pathways potentially participate in PF-mediated neuroprotection. Third, the core mechanistic evidence was obtained from rat-origin PC12 cells, and such PC12 cell and acute toxin-based in vivo models cannot fully recapitulate the complex pathological features of human Parkinson’s disease. Definitive in vivo pathway validation in mouse nigral tissues as well as verification in primary or human-derived neuronal models is still needed. Finally, the relatively low BBB penetration efficiency of PF implies that both central direct neuroprotection and peripheral indirect regulation, including gut microbiota modulation and systemic anti-inflammation, may jointly contribute to the therapeutic effects. Future work will focus on resolving the above limitations and optimizing PF structural modification to enhance CNS bioavailability, thereby promoting the translational application of PF as a promising natural neuroprotective agent for counteracting toxin-triggered neuronal injury.

## Data Availability

The original contributions presented in this study are included in the article. Further inquiries can be directed to the corresponding author(s).

## Abbreviations

Akt	protein kinase B
ANOVA	analysis of variance
BAD	Bcl-2-associated agonist of cell death
Bax	bcl2-associated X protein
BBB	blood–brain barrier
BCA	bicinchoninic acid
Bcl-2	b-cell lymphoma 2
BDNF	brain-derived neurotrophic factor
BP	biological process
cAMP	cyclic adenosine monophosphate
Caspase-3	cysteine-aspartic protease 3
CC	cellular component
CNS	central nervous system
CREB	cAMP response element-binding protein
CytoNCA	Cytoscape Network Centrality Analysis
DA	dopamine
DAVID	Database for Annotation, Visualization and Integrated Discovery
DCFH-DA	2′, 7′-dichlorofluorescein
DMEM	Dulbecco’s modified Eagle medium
DMSO	dimethyl sulfoxide
DNA	deoxyribonucleic acid
DOPAC	3,4-dihydroxyphenylacetic acid
DTT	dithiothreitol
ECL	enhanced chemiluminescence
ECM	extracellular matrix
ER	estrogen receptor
ErbB	erythroblastic leukemia viral oncogene homolog
ERK	extracellular signal-regulated kinase
FBS	fetal bovine serum
FC	fold change
FITC	fluorescein isothiocyanate
Fluo-3 AM	Fluo-3 acetoxymethyl ester
GO	gene ontology
HMGB1	high mobility group box 1
HO-1	heme oxygenase-1
HRP	horseradish peroxidase
HSP90AA1	heat shock protein 90 alpha family class A member 1
HVA	homovanillic acid
IL-1β	interleukin-1 beta
IL-6	interleukin-6
iPSC	induced pluripotent stem cell
Keap1	Kelch-like ECH-associated protein 1
KEGG	kyoto encyclopedia of genes and genomes
KOBAS 3.0	KEGG Orthology-Based Annotation System
LC-MS/MS	liquid chromatography-tandem mass spectrometry
LDH	lactate dehydrogenase
LPS	lipopolysaccharide
MAPK	mitogen-activated protein kinase
MDCK	madin-darby canine kidney
Mdr1	multidrug resistance protein 1
MEK	mitogen-activated protein kinase kinase
MF	molecular function
MPTP	1-methyl-4-phenyl-1,2,3,6-tetrahydropyridine
mTOR	mammalian target of rapamycin
MTT	3-(4,5-dimethylthiazol-2-yl)-2,5-diphenyltetrazolium bromide
NF-κB	nuclear factor kappa-B
Nrf2	nuclear factor erythroid 2-related factor 2
p90RSK	p90 ribosomal S6 kinase
PARP	poly(ADP-ribose) polymerase
PBS	phosphate-buffered saline
PC12	pheochromocytoma 12
PD	Parkinson’s disease
PF	paeoniflorin
P-gp	p-glycoprotein
PI	propidium iodide
PI3K	phosphoinositide 3-kinase
PVDF	polyvinylidene fluoride
Raf	rapidly accelerated fibrosarcoma
Ras	rat sarcoma viral oncogene homolog
ROS	reactive oxygen species
ROT	rotenone
RSK	ribosomal S6 protein kinase
S.E.M.	standard error mean
SDS	sodium dodecyl sulfate
SDS-PAGE	sodium dodecyl sulfate polyacrylamide gel electrophoresis
SIRT4	sirtuin 4
STAT3	signal transducer and activator of transcription 3
TCM	traditional chinese medicine
TGP	total glycosides of Paeonia lactiflora
TH-positive neurons	tyrosine hydroxylase-positive neurons
TLR4	toll-like receptor 4
TMT	tandem mass tag
TNF-α	tumor necrosis factor-alpha
TrkB	tropomyosin receptor kinase B
UniProt	Universal Protein Resource
UPLC	ultra-performance liquid chromatography
α-syn	alpha-synuclein
